# Women’s perceptions of telephone interviews about their experiences with childbirth care in Nigeria: A qualitative study

**DOI:** 10.1371/journal.pgph.0001833

**Published:** 2023-04-19

**Authors:** Nasir Umar, Zelee Hill, Joanna Schellenberg, Özge Tuncalp, Moise Muzigaba, Nuraddeen Umar Sambo, Abdulrahman Shuaibu, Tanya Marchant

**Affiliations:** 1 Department of Disease Control, London School of Hygiene & Tropical Medicine, London, United Kingdom; 2 Institute for Global Health, University College London, London, United Kingdom; 3 Department of Sexual and Reproductive Health and Research, UNDP/UNFPA/UNICEF/WHO/World Bank Special Programme of Research, Development and Research Training in Human Reproduction, World Health Organization, Geneva, Switzerland; 4 Department of Maternal, Newborn, Child and Adolescent Health and Ageing, World Health Organization, Geneva, Switzerland; 5 Data Research and Mapping Consult Limited, Abuja, Nigeria; 6 Office of the Executive Secretary, Gombe State Primary Health Care Development Agency, Gombe, Nigeria; Jhpiego, UNITED STATES

## Abstract

Our objective is to investigate women’s perceptions of phone interviews about their experiences with facility childbirth care. The study was conducted between October 2020 and January 2021, in Gombe State, Nigeria. Participants were women aged 15–49 years, who delivered in ten study Primary Health Care centres, provided phone numbers, and consented to a follow-up telephone interview about their childbirth experience. The phone interviews took place 14 months after the delivery and consisted of a quantitative survey about women’s experiences of facility childbirth followed by a set of structured qualitative questions about their experiences with the phone survey. Three months later 20 women were selected, based on their demographic characteristics, for a further in-depth qualitative phone interview to explore the answers to the structured qualitative questions in more depth. The qualitative interviews were analysed using a thematic approach. We found that most of the women appreciated being called to discuss their childbirth experiences as it made them feel privileged and valued, they were motivated to participate as they viewed the topic as relevant and thought that their interview could lead to improvements in care. They found the interview procedures easy and perceived that the call offered privacy. Poor network connectivity and not owning the phone they were using presented challenges to some women. Women felt more able to re-arrange interview times on the phone compared to a face-to-face interview, they valued the increased autonomy as they were often busy with household chores and could rearrange to a more convenient time. Views about interviewer gender diverged, but most participants preferred a female interviewer. The preferred interview length was a maximum of 30 minutes, though some women said duration was irrelevant if the subject of discussion was important. In conclusion, women had positive views about phone interviews on experiences with facility childbirth care.

## Introduction

The high coverage of mobile phones in low-and middle-income countries (LMICs) is advancing the prospect of using phone interviews to generate timely routine data on experiences of facility childbirth. This is needed by facility staff and managers to effect positive changes in childbirth care [1–4]. Mobile phones are increasingly utilised in disease surveillance, patient care and health promotion in many low-resource settings, with promising results [4–7]. The use of mobile phones to gather information about women’s experiences of facility-based childbirth care is however not yet mainstream.

The renewed interest in how women are treated during facility-based childbirth has highlighted the widespread and different forms of mistreatment that can occur [8–10]. Mistreatment during childbirth violates women’s fundamental human rights and may have a negative consequence on health outcomes and future health-seeking behaviour [11–13]. To address this, facility staff and managers require timely data on the frequency and forms of mistreatment occurring in their health facilities [14].

Progress has been made in developing tools to measure respectful and patient-centred care. However, most tools were developed for face-to-face administration, which requires more resources, including time to implement, than telephone interviews [15–18]. These surveys either sample women from the community, which can make linking data to specific facilities difficult, or are conducted at facility discharge when women may find it difficult to respond truthfully about their experiences. There is a need to assess how alternative methods (phone-based or computer-based technologies) could be used to provide facility-specific data that is collected in a more neutral setting and at a more convenient time than at facility discharge.

Although changing as a result of the need to use phone surveys in the COVID-19 pandemic, there has been a general reluctance for the wider use of phone interviews due to a perception that it is hard to establish rapport and impossible to read visual cues on the phone, and concerns about coverage bias [19]. An understanding of how long a phone interview should take or the level of complexity acceptable is limited [20]. However, the advantages of phone interviews described by de Leeuw and colleagues included how phone interviews may result in access to a wider range of hard-to-reach participants where phone ownership is high, and faster completion of phone interviews, 31 minutes (face-to-face interview) vs 24 minutes (telephone Interview) [21,22]. Cost advantages of telephone interviews due to a reduction in interviewer expenses (no travelling is necessary) have been reported [20,23,24].

Studies that compared face-to-face to telephone interviews reported no difference in output by mode of data collection, with the two methods yielding comparable data [25,26]. Others observed no difference in social desirability from participants responding to sensitive questions, though there are studies suggesting sensitive topics are more easily discussed on the phone due to the perception of control over own social space and a greater sense of anonymity [27,28]. Taken together, based on the literature reviewed, telephone interviews are a promising alternative or supplement to face-to-face interviews. Hence, it is plausible that phone interviews are a suitable method to talk to women about their experience of childbirth care. In this study, we qualitatively explored women’s perceptions and acceptability of telephone interviews about their experiences of facility childbirth care.

## Methods

### The study setting

The study was conducted in Gombe State, Nigeria. Gombe State has an estimated population of 3 million, the state is multi-ethnic and mostly rural: Farming, cattle-herding, and trading are the most common economic activity in the state; and an estimated 72% of its people live below $1 per day. Health services are provided primarily through government run primary health care facilities, general hospitals, one specialist hospital and the Federal Medical Centre, while private health services are provided by private clinics and private laboratory. Health service utilisation continues to be low, with just 44% of women with a recent live birth having four or more antenatal visits and just 28% giving birth in a health facility, and maternal, newborn and child mortality and morbidity continue to be high [9,29–31]. About 77% of households have mobile phone subscriptions in Gombe State [32].

### Sampling and data collection

Participants were part of a larger study investigating the validity of telephone interviews to collect data on women’s experiences with facility childbirth. Eligible participants were 388 women aged 15–49 years, who had childbirth in one of ten Primary Health Care centres between July and August 2019, who at discharge provided phone numbers and signed a consent form for a follow-up telephone interview and provided verbal consent before the commencement of the follow-up telephone interview. The follow-up telephone interview took place 14 months after discharge, 13–15 months recall has previously been used in similar criterion validity studies [33]. At the start of the phone interview, women were reminded of the background of the study and were asked if they were available for the interview. The survey consisted of quantitative questions about experiences with facility childbirth care [9]. Following the administration of the survey, all women were invited to remain on the call for 10–15 minutes to answer a set of structured qualitative questions about their experiences with the phone survey. The structured nature of the qualitative interviews, which did not include probing questions, ensured that every interviewee was asked the same questions. Topics included the acceptability of the telephone interview procedures, challenges of participation, what they liked or didn’t like, and whether they would be open to taking part in another telephone interview in the future.

Three months later, 20 of the women that participated in the structured qualitative telephone interview were selected, two women from each of the ten study health facilities, to participate in a more in-depth qualitative telephone interview (IDI) to gain deeper understanding of the women’s experience of the quantitative phone survey. Participants were purposively sampled to provide a range of demographic characteristics including age, educational level, and ethnicity that reflected the demographic composition found in the initial telephone interview sample. Women were asked detailed questions about their perceptions and experience of the quantitative phone survey on childbirth experience. For example, in the structured qualitative telephone interview women were asked what time they preferred to be called. In the in-depth interview, the question was expanded to understand why they prefer that time. The IDIs gave the interviewers the latitude to probe for more details and provided the respondents with the space to express their opinions, ask the interviewers questions, and give more in-depth information. The IDIs were conducted over the telephone and took approximately 15–30 minutes. All the women used the phones they owned or have access to for the telephone interviews.

All interviews were conducted by one of three female data collectors from Gombe State, who were trained in administering both the quantitative and qualitative instruments. Data were collected in English or Hausa depending on the respondent’s preference and all interviews were audio-recorded with the respondent’s consent. Measures to improve data quality included reviewing the interview recordings on a daily basis and holding debriefing sessions with interviewers to discuss areas for improvement or opportunities for probing. Measures to ensure data protection and respondents confidentiality included anonymising the data, transferring the data to password-protected study laptops, backing up the data on an external hard drive, storing the data in a locked drawer and restricting access to the final study dataset to only the research team members.

### Data analysis

The audio recordings from the structured qualitative telephone interviews were analysed directly based on the approach recommended by Halcomb and Davidson [34]. NU listened to the audio recordings to derive notes and then formulated codes and themes based on these notes. Thematic coding of the notes in Nvivo version v.13 followed and involved grouping related concepts to generate core themes. NU & NS then jointly reviewed, verified, and confirmed the findings.

IDIs were transcribed in English by the data collectors and checked for transcription and translation errors by NU. They were analysed using a thematic approach informed by Braun and Clarke [35]. After the transcripts were read for familiarity, they were coded into the same coding tree as the structured qualitative interviews with additional codes that emerged during the IDI analysis added inductively, resulting in one NVIVO file combining the structured qualitative interviews and IDIs. Combining the structured interviews and the IDIs aided the triangulation of findings. Combining deductive approach to organise our structured interview data and inductive approach that allowed additional codes to emerge during the IDI analysis reinforced the rigorousness of the analysis, while the thematic analysis allowed for data to be organised into themes, for enhanced description of respondent’s experiences and perceptions [35]. The study findings are reported in line with the Consolidated Criteria for Reporting Qualitative Research (COREQ) [36] and STROBE (S1 Checklist).

### Ethics approval

The study was reviewed and approved by the Federal Ministry of Health Abuja, Nigeria (reference NHREC/01/01/2007), the Gombe State Ministry of Health, Nigeria, and the London School of Hygiene & Tropical Medicine (reference 12181). To obtain participant’s signed consent at the time of discharge, information about the study, participant’s right to participate or refuse to take part in the study, their right to end their participation at any time during the study, and how the information they provided will be used and shared, how their confidentiality and anonymity will be maintained was read and explained to all potential participants before they exit the health facility, and were also provided with an information sheet to take home. We obtained informed consent from parents or guardians of participants less than 18 years of age (minors) included in the study. During the follow-up telephone interview, information about the study was once again read and explained to all potential participants. Only those that provided verbal consent were interviewed.

### Patient and public involvement

Prior to the main telephone interviews, we conducted preliminary consultation with a different set of women to pre-test the telephone interview protocol for appropriateness and understanding. Respondents were asked for feedback about the telephone interview procedures including perceived difficulty, compatibility, and clarity of instructions. The telephone interview protocol was refined and finalised based on the respondent’s inputs. Information was given to the respondents on their right to answer the phone or schedule the phone interview, but not on how to use of the phone.

### Inclusivity in global research

Additional information regarding the ethical, cultural, and scientific considerations specific to inclusivity in global research is included in the (S1 Text).

## Results

### Participant’s characteristics

A total of 388 women provided phone numbers at the time of discharge following facility childbirth and signed a consent form for the follow-up telephone interview, of whom 294 (76%) were reached via telephone. All the 294 women eligible for the structured telephone interview participated. The majority were married (99%), between the ages of 20–29 years (64%), and 39% had no formal education. Similarly, the 20 women participating in the in-depth interviews were predominantly married (95%), between the ages of 20–29 years of age (60%), and 25% had no formal education (Table 1). None of the 20 women invited for the IDIs refused to participate.

**Table 1:** Characteristics of the study sample.

	Structured qualitative telephone interviews (N = 294) N (%)	In-depth telephone interviews (N = 20) N (%)
Age of woman at delivery		
15–19	31 (11)	-
20–24	102 (35)	6 (30)
25–29	85 (29)	9 (45)
30–34	44 (15)	3 (15)
>34	32 (10)	2 (10)
Educational attainment		
None	114 (39)	5 (25)
Primary	60 (20)	4 (20)
Secondary	106 (36)	9 (45)
Higher	14 (5)	2 (10)
Marital status		
Married	292 (99)	19 (95)
Not Married	2 (1)	1 (5)
Religion		
Islam	281 (96)	16 (80)
Christianity	13 (4)	4 (20)

We identified eight overarching themes from the structured telephone interview and IDIs, conducted to describe women’s perceptions about the telephone interview including 1) feeling privileged and valued to participate in the telephone interview; 2) motivation for participation in the telephone interview; 3) ease of participation in the telephone interview; 4) privacy and confidentiality of the telephone interview; 5) autonomy of participation in the telephone interview; 6) scheduling and convenience of participation in the telephone interview; 7) gender preference for the telephone interviewer; 8) duration of the telephone interview.

#### Theme 1. Feeling privileged and valued to participate in the telephone interview

Reacting to being called to participate in the quantitative telephone interview about their experiences with facility childbirth care, participants described feeling privileged for being chosen and called from among many mothers to talk to about their experience of facility-based childbirth care. They felt valued that someone cared enough to call and talk to them. Women expressed their surprise that the call happened at all, they mentioned providing their phone numbers at the health facilities in the past, but without receiving any follow-up calls from the health facilities.

“I felt privileged that I was called…I am happy to be part of the solution to improve care for women.” (27 years, #030)

“It [the telephone interview] shows that they care about our health…what impressed me was that among all my deliveries only in this third one that I was called and asked about my care and that of Muhammed (my son)–that really impressed me.” (IDI participant, 22 years, #067)

“When they [facility staff] asked for my number at the health facility I asked them why, they told me they want to call me and find out about the care I received during childbirth, I didn’t believe it will happen, but I gave the number anyway, so I was pleasantly surprised someone called.” (IDI participant, 36 years, #042)

#### Theme 2. Motivation for participation in the telephone interview

Motivation for participation in the telephone interview was another common theme identified. Women attributed their willingness to participate to the fact that the telephone interview offered an easy way to talk about facility-based care. They considered the interview subject and the questions asked to be very important and relevant to them, and suggested it was their responsibility to respond. They considered the telephone interview about their childbirth care now, or in the future, as being beneficial to women and could lead to change in health worker practices.

“Yes, what they can’t do at hospital I will tell you at home….it is good way to find out what is not happening at the facility.” (IDI participant, 30 years, #003)

“The interview was about bringing improvement and progress for us to benefit from…any health worker that knows that questions are being asked about the care women received during facility birth…I believe they will improve their practices and habit if they want to keep their job.” (IDI participant, 36 years, #042).

#### Theme 3. Ease of participation in the telephone interview

The ease of participation was frequently mentioned by the participants. They described it in terms of not needing help to use the phone and the easy rapport with the interviewer. They mentioned how they enjoyed the discussion or how the telephone interview was like a regular discussion to them. The women attributed the ease of participation to being habitual users of mobile phones. Notwithstanding the ease of participation with which women found the telephone interviews, some women highlighted as a limitation the lack of physical interaction, and not seeing the person they were talking to, that was associated with the telephone interview.

“I did not need help…you ask for help for something you don’t know about…” (IDI participant, 34 years, #009)

“The only difference is…[in] phone interviews you only make calls, without seeing each other face to face, and you will not know the person calling you.” (IDI participant, 28 years, #010)

Challenges to participation included poor network and connectivity issues on some days, or low phone battery power due to infrequent electricity. The women acknowledged neither the interviewer or the interviewee could do anything regarding the connectivity issues other than to wait and call back later or charge phones in advance of the interview.

“Just like what you did [when the network was poor], send a text message when the network is not good…and reschedule [the telephone interview] … it is not all the time that the network is problematic.” (IDI participant, 36 years, #042)

About one-third of the women reported not having their own mobile phone and rather relied on their spouses or relative’s phones. But it was neither a bother for the owners nor a hassle for them to participate. They said that their participation in the telephone interview was dependent on the owner (spouse or relative) being at home or close by, regardless of their spouses or relative’s willingness to let them use their mobile phones with no restriction. Participants pointed to arranging a call back with the owners for when they were back home or were in relative proximity with the potential participant as key to their participation.

“I was not bothered, because he [my husband] gave me the phone and left me with it…so no problem, because he doesn’t mind.” (IDI participant, 25 years, #035)

“It was my sister’s phone, and she will not ask for her phone back [when I have a call] …also I was not the one that called… [no cost to calls received].” (IDI participant, 24 years, #012)

“…the best time [to call me] would be when the owner is around, at home… mornings are better, you see the phone is not mine… the owner may need it in the afternoon.” (IDI participant, 27 years, #025)

#### Theme 4. Privacy and confidentiality of the telephone interview

Many participants stated their appreciation for being able to speak when they were alone or with minimal intrusion. Those that appreciated the perceived privacy offered by the telephone interview considered information about their delivery as their secret and their answers personal and confidential. However, not all the women shared this view, some of the women found nothing compromising talking about their experiences with facility childbirth care. Privacy and confidentiality during the telephone interview appeared not to depend on whether the respondents were using their own or a borrowed phone.

“…there was also privacy [with phone] …as it is between you and I alone.” (IDI participant, 34 years, #009)

“No, no one was listening…even my husband went out and left me.” (IDI participant, 25 years, #035)

“…even if someone heard the conversation there was nothing compromising.” (IDI participant, 24 years, #012)

#### Theme 5. Autonomy of participation in the telephone interview

A characteristic of the telephone interview mentioned by the participants was that over the phone they could decide to take the call or not, to participate or not, and the autonomy to tell the caller to call back later. Such autonomy they felt was not necessarily possible during a face-to-face interview when the interviewer was at the doorstep, because it is more difficult to say no when the interviewer was right in front of the women.

“There is no problem with phone call, if I have the time we discuss, if I don’t, I will say I don’t have the time…when someone [data collector] comes to your house…you will not feel at ease to say come back another day or time… but, with phone you could.” (IDI participant, 20 years, #008)

#### Theme 6. Scheduling and convenience of participation in the telephone interview

A recurrent theme among participants was the convenience they associated with the telephone interview. Household chores and economic activities like going to the farm or market all constitute an important part of the participant’s daily routine. Therefore, the interview timing was important: many women stressed that they might not have been able to participate at certain times of the day. Consequently, participants found the telephone interview convenient because they could schedule the call around their daily chores.

“When you wake up in the morning you will bathe your children, clear their rooms, prepare breakfast for them and then take them to school…and then comes lunch and dinner…then take your bathe and call off for the day.” (IDI participant, 22 years, #067)

“… when she called it was during raining season, face to face interview will not be convenient, because I go farm.” (IDI participant, 34 years, #007)

The preferred time for interview differed but mostly related to when the respondents were through or had a break with their household chores or work or job commitments, with some stating a preference for 8.00am-10.00am, some 1.00pm-2.00pm, others 7.30pm-8.30pm.

“[preferred time for the telephone interview] …when you are done with breakfast and lunch, but yet to start preparation for dinner.” (IDI participant, 27 years, #030)

#### Theme 7. Gender preference for the telephone interviewer

The gender of the person conducting the interview appeared to be important, as the purpose of a male interviewer calling them could be misunderstood by their husbands. Consequently, the majority of the respondents expressed strong preference for a female interviewer to avoid potential marital problems.

“The interviewer was a female, I don’t think I can talk to a man about my pregnancy and delivery, I am not his wife. In fact, I can’t discuss delivery issues with him. If I pick the call and discovered the call to be from a man, I will not answer the call…I will disconnect the call… as this can cause problem [marital] for us….” (IDI participant, 20 years, #008)

“If a man called……not my husband, I would not answer, because I need my husband’s permission to talk to another man.” (IDI participant, 25 years, #035)

Some women suggested that the gender of the telephone interviewer was less of an issue as long as the caller asked for permission from their husband. Husband’s trust has been mentioned as an enabler. The caller being a health worker was said to be enough for some of the women. To improve participation and buy-in from spouses, women recommended raising awareness for subsequent telephone interviews at the facility and at the community level.

“I will not have any problem taking calls from a male, though I can understand it is not the same for every woman, it depends if your husband’s trusts you.” (IDI participant, 36 years, #042)

“No problem [if the caller is male], because he is a health worker, and wherever you go, if it happens that it is only the man that can do the interview there wouldn’t be any problem.” (IDI participant, 22 years, #067)

#### Theme 8. Duration of the telephone interview

Following their participation in the follow-up telephone interview about their experiences with facility childbirth care which took approximately 45 minutes to complete, participants suggested that the telephone interviews should range between 10 minutes to 30 minutes, and not more than 30 minutes in general. However, there were some divergences, as some women suggested that the duration of the telephone interview did not bother them or would not be an issue if the subject of the interview or discussion was important and relevant to them.

“For us women, once children are around or awake, our concentration is limited. It is important to make the interview as short as possible, like 15 minutes, so that the respondent does not get tired, and would be happy to participate in the interview next time another interviewer call.” (IDI participant, 30 years, #008)

“I cannot say the number of minutes it [telephone interview] should take…in my opinion, if the discussion is with me and I don’t have anything to do even if it will take ten hours, I don’t have problem…you give your time on important things…things that are not important even one minute make no sense.” (IDI participant, 24 years, #012)

## Discussion

Telephone interview methods have been effectively used to elicit health-related information in high-income countries for decades but application in LMICs is less common. Hence, the dearth of evidence on women’s perceptions and acceptability of telephone interviews about their experiences with facility childbirth care in LMICs. This study of telephone interviews in north-eastern Nigeria revealed that participants were overall accepting of telephone interviews to derive maternal self-report of experiences with facility childbirth care, highlighting barriers and enablers to acceptability captured in eight themes. The themes included feeling privileged and valued, motivation for participation, ease of participation, privacy and confidentiality, the autonomy of participation, scheduling and convenience of participation, gender preference for the telephone interviewer, and duration of the telephone interview.

While we could not identify any prior studies from which to draw direct comparisons, the use of mobile phones has been investigated in other related health fields in LMIC with results that resemble some of our findings [37,38]. Mobile phones have been used to deliver interventions, for research and data collection in LMICs such as Bangladesh, Honduras, Brazil, Lebanon, Mali, Peru, South Sudan, Tanzania, Afghanistan, Ethiopia, Kenya, South Africa, Zimbabwe, for a wide range of topics including mental health, HIV/AIDS, sexual and reproductive health, adolescence health, drugs use, and non-communicable [6,37–39]. Studies that explored acceptability reported varying, but mostly high acceptability of mobile phone use in interventions, research or as data collection platforms [6,39]. Similarly, participants in this study were accepting of mobile phone-based interviews. Perhaps indicating a general trend in LMICs, though the characteristics driving the acceptability may vary.

The perceived relevance of the telephone interview to lead to some improvements was a driver of women’s participation. In telephone interview, interviewer skill and the information provided to the potential participant are considered key to high participation [40]. In our study, women were provided with information about the study during recruitment at the health facility and given information sheet to take home for further consultation. Perhaps, aiding the respondents greater understanding of the objectives of the study, and respondents’ attribution of their motivation to participation to the perceived relevance of the telephone interview. Additional unique findings in this study are the respondents felling privileged and valued for being chosen and called from among many mothers, and the autonomy of participation, which could cautiously be interpreted as signalling the potential for wider acceptance of phone base interviews about women experience of childbirth care. One of the characteristics enabling the acceptance of the telephone interview among the study participants was that they found the telephone interview easy. Participants were only required to answer the phone call and respond to questions. Being habitual mobile phone users, even among those who relied on their spouses, relatives or friend’s phones, participants found the interview easy to participate in. Participant’s perceived ease to use a technology has been associated with increased acceptability [41,42]. The study participants highlighted the ability to schedule the interview at a time convenient to both the interviewer and the interviewee as an advantage of the telephone interview, and enabler of acceptability. An earlier study has pointed to a direct relationship between convenience and increased acceptability especially by older adults [43]. In few studies there are divergent findings regarding time and day preferences for phone-based surveys [41,44], but the option of data collectors speaking to interviewees to schedule convenient interview times appears to offer a favourable approach. Many participants stated their appreciation for the perceived privacy offered by the telephone interview, in relation to being able to speak when they were alone or with minimal intrusion. However, to the few of the respondent’s, privacy was irrelevant. Perception of privacy and confidentiality was not related to whether the phone used for the interview belonged to the respondents or not, contrary to our expectations. There is currently limited evidence in the literature about perceptions or expectation of privacy and confidentiality during telephone interviews, or on the effects of approaches to improve phone interview privacy and confidentiality in LMICs [6,41].

An important enabler or a potential barrier to acceptability of the telephone interview was the interviewer gender preference. Most of our study participants were married and most would prefer a female to a male interviewer, to avoid marital problems. A near comparable study relied on automated voice calls, with female participants in the study preferring female to male voice, to avoid violence at home, while some of the male participants would prefer a female voice [41]. As more remote interviews or surveys become popular in LMICs post COVID-19, our findings reinforce the need for more studies to investigate interviewer gender preferences for in-person or voice modes, in different contexts. Reflecting on their experience of participation, respondent’s comments about the length of the telephone interviews showed that there is a limit to how long participants were willing to answer questions over the telephone, with 10–30 minutes as the most cited. Though this view was not universal, some respondents would be willing to have a longer interview if the subject of discussion is relevant to them. Studies including this study have reported varied perceptions about what respondents thought would constitute appropriate time for a phone interview [38,44,45]. Telephone interview respondents are more likely to complain about the phone interview duration than respondents in face-to-face interview, possibly owing to the social distance and the different dynamics between the two data collection modes [38,44].

Limited phone ownership in LMICs has previously been a concern when developing phone-based approaches, as low coverage means fewer people can be accessed and population sub-groups cannot be appropriately represented [21,41]. However, our study is consistent with other reports that not owning a phone does not necessarily mean not having access [37,39]. Participants in this study were able to engage in the interview using mobile phones belonging to their spouse, relative or a friend. Another barrier to acceptability cited by the study participants was poor network connectivity (when it happens). Poor network connection or network challenges have been mentioned in studies from similar settings [37,39,41] Our study participants acknowledged this reality, suggesting one way to address the situation would be to wait for the network to improve.

## Implications

This study identified critical areas that could improve the prospects of phone-based engagement or interventions in LMICs. Our findings suggest that women found the interaction easy to use and service providers could consider engaging with service-users by phone to receive feedback [6,39]. Regarding design, our study illustrates the importance of appropriateness of engagement to cultural sensitivities, as some of our study participants considered it more appropriate for a woman to be interviewed by a fellow woman. Our study also suggests that domestic or economic activities of the respondents could have an impact on the acceptability of phone-based engagement or interventions and should be considered during both the design and evaluation of phone-based engagement or interventions in LMICs.

Our study has its strengths and limitations. We utilised well-trained researchers from Gombe state, the study setting, who were familiar with the setting and attuned to the culture and context, which may have improved respondent’s ease of participation and building rapport, as well as mitigating the risk of the researchers inadvertently imposing their beliefs, values, and patterns of behaviour upon the respondents and the cultural settings in which the study was conducted [46,47]. The data collectors had no prior engagement with the respondents, mitigating the risk of coercion to participate or respond to interviews [48]. Data quality was assured by reviewing the interview recordings daily and holding debriefing sessions with interviewers and ensuring the appropriateness of transcription and translation. Our chosen analysis approach, thematic analysis, is theoretically flexible and enabled us to synthesise our data both inductively and deductively [49]. The approach allowed us to focus our unit of analysis on identifying and describing both implicit and explicit ideas and reporting patterns (themes) within data rather than the more micro-level focus of counting explicit words or phrases used, their repetition, and relationships that characterised content analysis [35,50]. We sought to organise and describe our data in rich detail to investigate women’s perceptions of phone interviews about their experiences with facility childbirth care, and not to generate a plausible theory that would otherwise require a different analysis approach, such as grounded theory analysis. However, thematic analysis has its limitations. For example, the approach could be seen to have limited interpretative power beyond mere description, and unlike the narrative approach, retaining a sense of continuity and contradiction through any one woman’s account is not possible, and these contradictions and consistencies across individual women’s accounts could be revealing [35]. The participants were women who had a facility-based childbirth in selected facilities and who had provided numbers for phones they owned or had access to, for the telephone interview. Women without access to a phone or who never had a facility childbirth may experience a telephone interview differently, this may be particularly true in the study setting where the majority of births are at home. We evaluated quantitative telephone interviews through qualitative telephone interviews, it could be argued that the responses obtained from the participants in the qualitative telephone interviews might be shorter and less in-depth, due to limited ability to probe. Our study relied on self-report of women’s perceptions and acceptability following participation in the telephone interview; the same interviewers conducted the phone survey as well as asked women about their experiences with participation, hence, there was potential for social desirability bias. We had originally planned for a shorter interval for the follow-up interviews, but the original study plans were disrupted by the COVID-19 pandemic: we cannot know what effect 17 months recall period had on women’s ability to remember certain issues, nor discount the likelihood of recall bias. Yet, this study provides new insight on the potential of using telephone interviews to talk to women about their experiences with facility childbirth care in LMICs.

## Conclusions

In a predominantly rural setting in north-eastern Nigeria, we found that women attending facility childbirth care perceived mobile phone interviews to be acceptable, citing the importance of the topic, the convenience of the method, and enhanced autonomy and privacy amongst the positive characteristics. Despite their potential, acceptability cannot be assumed. More qualitative studies to understand contextual enablers and barriers would aid acceptability of phone base interview or interventions, as highlighted by the study findings presented here.

## Supporting information

S1 Checklist Strengthening the reporting of observational studies in epidemiology checklist contained items that should be included in reports of observational studies. (DOCX)

S1 Text Policy on inclusivity in global. (DOCX)
